# Karyological Diversification of *Trochoidea caroni* (Gastropoda, Pulmonata, Geomitridae) Between Sicilian and Non-Sicilian Populations

**DOI:** 10.3390/ani15172596

**Published:** 2025-09-04

**Authors:** Agnese Petraccioli, Gaetano Odierna, Paolo Crovato, Fabio Maria Guarino, Rosa Carotenuto, Ignazio Sparacio, Nicola Maio

**Affiliations:** 1 Dipartimento di Biologia, Università di Napoli Federico II, Via Cintia 26, 80126 Naples, Italy; petra.ag@gmail.com (A.P.); gaetano.odierna@unina.it (G.O.); rosa.carotenuto@unina.it (R.C.); nicola.maio@unina.it (N.M.); 2 Società Italiana di Malacologia, Via Mezzocannone 8, 80134 Naples, Italy; paolo.crovato@fastwebnet.it; 3 Independent Researcher, Via Principe di Paternò 3, 90144 Palermo, Italy; edizionidanaus@gmail.com

**Keywords:** karyotype, NOR-FISH, chromosome evolution, *Trochoidea*, land snails

## Abstract

*Trochoidea caroni* (Gastropoda, Geomitridae) is a land snail previously found only in Sicily and Capri (Naples), but it has recently been found in other regions of the Italian peninsula. In this study, we performed karyological and molecular analysis on *T. caroni* from different sites across this regional range. Karyological analysis was performed on specimens from Palermo (Sicily), Capri (Campania), and Terracina (Lazio) using standard staining and NOR-FISH methods; the latter method was also performed on samples of *T. elegans* from Rome (Lazio). All *T. caroni* specimens had 2*n* = 48 chromosomes, but the 8th and 17th pairs differed morphologically between specimens from Capri and Terracina and those from Sicily. The mitochondrial 16S rRNA analysis grouped Sicilian and non-Sicilian populations of *T. caroni* in distinct subclades. Superimposing karyological data on their phylogenetic tree suggests that possible chromosomal rearrangements occurred during the diversification of *Trochoidea*. Our findings provide karyological and molecular evidence for a diversification between Sicilian and non-Sicilian populations of *T. caroni*. Furthermore, NOR-FISH revealed hybridisation signals on the 16th chromosome pair in both *T. caroni* and *T. elegans* (tribe Trochoideini). Similar NOR localisation has also been identified in *Cernuella virgata* (tribe Cernuellini), suggesting that it was inherited from the common ancestor of Trochoideini and Cernuellini.

## Simple Summary

Land molluscs are one of the main contributors to global animal diversity, particularly to European fauna. Among Italian land gastropods, *Trochoidea caroni* (Deshayes, 1832) and *T. elegans* (Gmelin, 1791) are some of the most biogeographically interesting species. In fact, they belong to a group of species (“*elegans* group”) whose systematic position remains to be fully defined, despite being considered valid by most authors. *T. caroni* is currently an endemic species of the Italian peninsula and Sicily, while *T. elegans* has a western circum–Mediterranean distribution, but it was introduced to Great Britain and is rare in Belgium. It is not yet possible to draw certain conclusions on the unusual biogeography of these taxa, but our karyological data improve current knowledge on the phylogeography of *T. caroni*, and on the possible origin and evolution of these two taxa. We propose an evolutionary scenario for the chromosome rearrangements that occurred during the specific diversification of *Trochoidea.* Furthermore, the presence of NOR loci on the 16th pair of chromosomes in both Trochoideini and Cernuellini tribes suggests their derivation from a common ancestor.

## 1. Introduction

Geomitridae C. R. Boettger, 1909, is a widespread family of small- to medium-sized terrestrial snails distributed in northern Eurasia, the eastern Atlantic islands, North Africa, Madeira, the Azores, and the Canary Islands (Schileyko, 2006). The family Geomitridae family recently separated from the Hygromiidae (Razkin et al., 2015), includes numerous species (about 400) divided into 51 genera and 2 subfamilies—Geomitrinae C. R. Boettger, 1909, and Helicellinae Ihering, 1909—both of which consist of various tribes. The Geomitrinae family consists of the Cochlicellini Schileyko, 1972; Geomitrini C. Boettger, 1909; and Ponentinini Schileyko, 1991, tribes. Helicellinae Ihering, 1909, includes the following tribes: Cernuellini Schileyko, 1991; Helicellini Ihering, 1909; Helicopsini H. Nordsieck, 1987; Plentuisini Razkin, Gómez-Moliner, Prieto, Martínez-Ortí, Arrébola, Muñoz, Chueca and Madeira, 2015; and Trochoideini H. Nordsieck, 1987 [1].

*Trochoidea* Brown, 1827, is an interesting genus of the latter tribe, comprising both narrowly distributed species (also endemics) and forms with relatively wide distribution. According to Molluscabase [1], the following species are attributed to this genus: *Trochoidea elegans* (Gmelin, 1791), which is distributed in several European countries and North Africa (Spain, France, Italy, and Greece, and introduced in Great Britain, Tunisia, and Algeria); and *T. pyramidata* (Draparnaud, 1805) and *T. trochoides* (Poiret, 1789), which are widespread around the Mediterranean sea. The endemic taxa are as follows: *T. cucullus* (E. von Martens, 1875) and *T. spratti* (L. Pfeiffer, 1846), present in Malta; *T. cumiae* (Calcara, 1847), and *T. liebetruti* (Albers, 1852), living, respectively, in Lampedusa Island and Cyprus; *T. hipponensis* (Morelet, 1858), present in Algeria; *T. hesperidum* (Morelet, 1880) in Morocco; and *T. caroni* (Deshayes, 1832) and *T. tarentina* (L. Pfeiffer, 1848), endemic in Italy.

In Italy, the distribution area of the various taxa of *Trochoidea* is not clearly defined, except for that of *T. caroni*, locus typicus Sicily [2], previously reported as living in central-western Sicily and Capri [3,4,5]. New reports of living *T. caroni* specimens or their shells have recently been found in Latium, Tuscany (Museum specimen), and Campania, documenting a wider distribution of this taxon, unlike that previously hypothesised [6]. According to Bodon et al. [5], *T. caroni* has been introduced to peninsular Italy. Furthermore, the taxonomic status of various forms of *Trochoidea* is debated, particularly the ‘*elegans*’ species group, which includes *T. elegans*, *T. caroni*, *T. spratti*, and *T. cumiae* [7]. Molecular and karyological analyses are also largely unexplored in *Trochoidea*, providing relationships and karyotypes only of *T. elegans*, *T. pyramidata*, and *T. trochoides* [8,9,10,11].

Cytogenetic inferences, particularly when coupled with molecular data, have proven to be useful tools for identifying plesio- and apomorphic states and the presence of different evolutionary lineages and reproductive barriers, and for reconstructing the evolutionary trends of the studied taxa, including Gastropoda [12,13,14,15,16,17].

This paper presents the results of a karyological study conducted on *T. caroni* from different sites within its range, including Sicily (Palermo and Trapani) and sites outside of Sicily (Capri in Campania; Terracina in Lazio). Our findings were compared with those available in the literature concerning phylogenetically closely related species. The presence and distribution of primitive and derived chromosomal features are also discussed, and a proposal for the chromosomal diversification of *Trochoidea* is made. Preliminarily, to detect possible molecular diversification between Sicilian and non-Sicilian *T. caroni* populations, we also analysed a mitochondrial 16S rRNA gene fragment and performed a phylogenetic analysis that included other closely related *Trochoidea* species [8,9,10,18].

## 2. Materials and Methods

For karyological analysis, we used tissue samples of live *T. caroni* individuals: three samples from Capri (Naples, Campania, Italy), two samples from Terracina (Latina, Latium, Italy), four samples from Capaci (Palermo, Sicily, Italy), and two samples from Macari (Trapani, Sicily, Italy). All samples had already been analysed by Maio et al. [6], who discriminated them according to Giusti et al. [7], whose article details the origin of the specimens studied herein. For NOR-FISH staining, we also used chromosome samples of *T. elegans* from Santa Severa (Rome, Italy) that had already been used in our previous study [11]. No animals were sacrificed for this study.

### 2.1. Chromosome Analysis

Chromosomes were obtained as described in [11,14]. Briefly, gonads were incubated for three hours in 1 mL of calf serum containing 3 μg of a colcemid solution (10 μg/mL) and incubated for 30 min in hypotonic solution (0.35% sodium citrate solution + 0.28% KCl solution). Then, the gonads were fixed in 3:1 methyl alcohol + acetic acid for 15 min and scraped on a 100-mesh sieve. Cell suspensions were centrifuged at 1000 rpm and, after fixative refreshment, stored at −20 °C. If necessary, the cell suspensions were left at room temperature, and aliquots of 20 μL were dropped onto slides, air-dried, and stained for 5 min with 5% Giemsa solution at pH 7. Karyotypes were obtained from 5 enlarged metaphase plates, and the relative morphometric parameters of chromosomes were independently calculated by five authors (AP, NM, RC, FMG, and GO) using the public domain software Image J 1.45 (https://imagej.net/ij/, accessed on 1 June 2025). For chromosome nomenclature, we followed the work of Levan et al. [19].

NOR-FISH staining was performed as described by Petraccioli et al. [13] using the 18S rRNA biotin gene sequence units of the pectinid *Adamussium colbecki* (E. A. Smith, 1902) as a probe. After being dropped onto slides, the cells were aged for one day at room temperature, allowed to stand for two hours at 60 °C, and then incubated for 30 min in RNase at 100 μg/mL in Tris-HCl 10 mM at pH 6.5. Finally, the cells were dehydrated in alcohol series and air-dried. The chromosomes and probe were denatured for three min at 72 °C in the hybridisation mixture (10 ng/mL biotinylated 16 dUTP probe + 0.1 mg/mL shared E. coli DNA in 2× SSC with 50% formamide). The hybridisation was carried out at 40 °C for 20 h. Washes were performed in 1× SSC at 72 °C for 5 min and at RT for 2 min.

Probe detection was performed using monoclonal anti-biotin antibodies (Sigma cod. B7653) diluted 1:500 in PTB (1 mL PTB = 5 μL Tween 20% + 0.01 g dry milk in 1 mL 0.2 M PBS). After one hour, the slides were washed in 1 × PBS and incubated for 30 min in FITC-conjugated anti-anti-biotin antibodies (Sigma, Kawasaki, Japan) diluted to 1:50 in PTB. After washing in 1× PBS, chromosomes were counterstained with 5 μg/mL propidium iodide (PI) in 1× PBS for 15 min at RT and mounted with anti-fade (DABCO, Sigma). The hybridisation signals were detected and recorded under an epifluorescence microscope (Leica DM) equipped with a digital camera.

### 2.2. Molecular Analysis

DNA was extracted from the foot of two specimens for each studied population, according to Sokolov [20]. In brief, a foot piece (3–5 mm) from a specimen of each studied population was sliced with forceps. Tissue fragments were transferred to a 2 mL plastic tube containing 1 mL of the lysis buffer (50 mM Tris-HCl, pH 7.5, 100 mM NaCl, 10 mM EDTA, 1% sodium dodecyl sulphate (SDS), 0.2 mg/mL Proteinase K) and incubated at 55 °C until complete digestion. Successively, 100 μL of saturated KCl solution was added, incubated on ice for 5 min, and centrifugated at 12,000 rpm. The supernatant was extracted twice with an equal volume of chloroform/isoamyl alcohol (24:1) mixture. Finally, DNA was extracted by adding to the supernatant 2 volumes of absolute alcohol, washed in 70% alcohol, centrifugated a 6000 rpm, air-dried and, dissolved in an adequate volume of TE buffer (10 mM Tris-HCl, 1 mM EDTA) to finish.

The mitochondrial 16S rDNA fragment was amplified via polymerase chain reaction (PCR) using the same primer pairs and PCR conditions as used by Sá-Pinto et al. [21]. After purification using the QIAEX gel purification kit (Quiagen, Hilden, Germany), the PCR products were sequenced using the BigDye Terminator Cycle sequencing protocol on an ABI Prism 310 automated sequencer (Applied Biosystems, Waltham, MA, USA), and all sequences were submitted to GenBank (accession number PX119029-36). Sequence chromatograms were edited with ChromasLite© 2.6.4, aligned using ClustalW [22] in BioEdit 7.0.5.3 [23], and blasted in GenBank. A neighbour joining phylogenetic tree of 16S rDNA was inferred from the alignment using MEGA11 v11.0.13 [24] with 1000 replicates using the sequence of *Trochoidea* retrieved from GenBank and using the 16S rDNA of *Albinaria caerulea* (A,N: NC_001761) as an outgroup. The 16S rDNA sequences of *Xerocrassa* (formerly *Trochoidea*) available in GenBank were not included because they were shorter than the other examined sequences.

## 3. Results

### 3.1. Karyological Analysis

**Figure 1:**
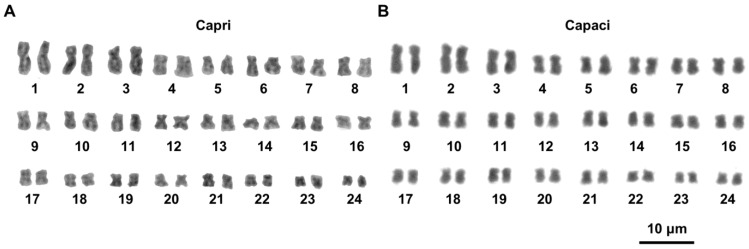
Giemsa-stained karyotypes of *T. caroni* specimens from Capri (**A**) and Palermo (**B**). The scale bar applies to both images.

Metaphase plates suitable for chromosome analysis were obtained from specimens from Capri, Terracina, and Palermo, which showed a karyotype of 2*n* = 48 chromosomes, with the first three pairs distinctly longer than the others, and which progressively decreased in length (Figure 1).

Specimens from Capri and Terracina had very similar karyotypes, with the metacentric pairs 1–2, 4–6, 9–11, 13, and 15–24; submetacentric pairs 3, 7, 12, and 14; and the subtelocentric pair 8. The karyotype of Palermo specimens differed from that of Capri/Terracina in showing metacentric chromosomes in the 8th pair and submetacentric elements in the 17th pair (Figure 1; Table 1).

**Figure 2:**
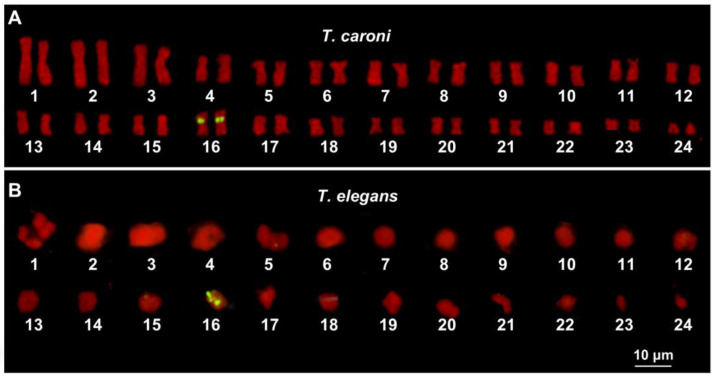
Karyotypes from the gonadic metaphase (**A**) and diakinetic meiotic plates (**B**) of *T. caroni* (**A**) and *T. elegans* (**B**). The scale bar applies to both images.

In *T. caroni*, NOR-FISH was performed on metaphase plates, while in *T. elegans*, only diakinetic meiotic bivalents were suitable for the analysis. In the former, clear signals of hybridization were identified close to the centromeres of the short arms of 16th chromosome pair. In *T. elegans*, hybridization signals were detected on a bivalent presumed to be the 16th pair (Figure 2A,B).

### 3.2. Molecular Analysis

**Figure 3:**
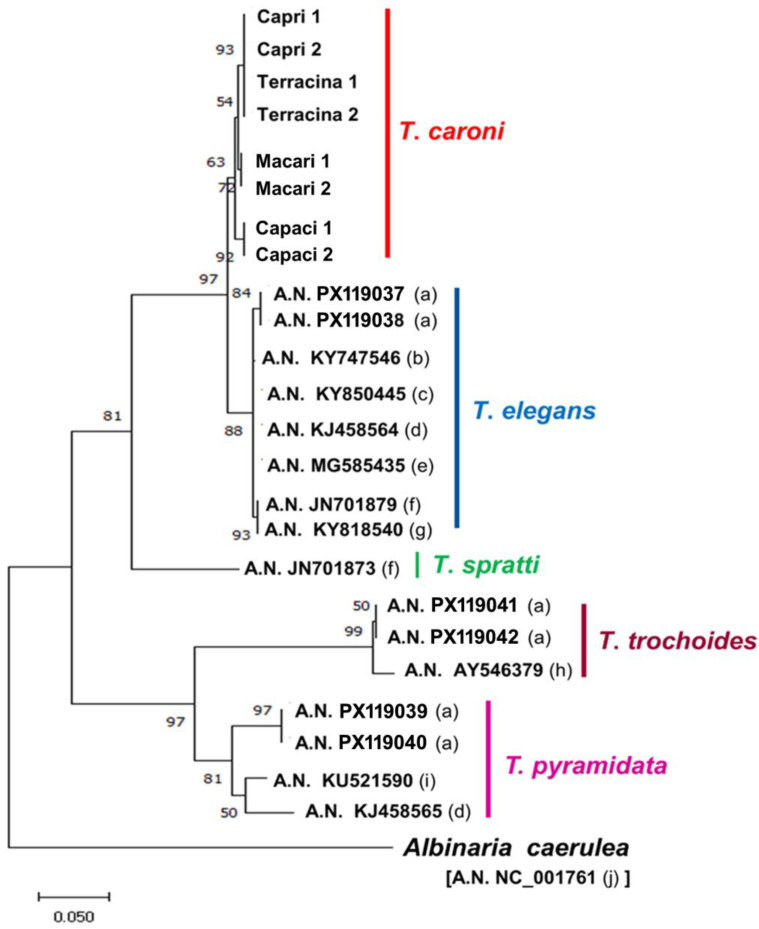
16S rDNA neighbour joining phylogenetic tree showing the relationships between the studied samples of *T. caroni* and the deposited homologous GenBank segments of other *Trochoidea* species. a = present study; b = Ezzine et al. [25]; c = Chueca et al. [26]; d = Razkin et al. [10]; e = Caro et al. [27]; f = Sauer and Hausdorf [28]; g = Neiber et al. [29]; h = Steinke et al. [9]; i = Boeckers et al. [30]; j = Hatzoglou et al. [31].

The selected fragments of 16S were successfully amplified in the study samples, and the newly generated sequences were deposited in GenBank (accession numbers PX119029 to PX119036). The Nj and ML phylogenetic analysis of the *T. caroni* sequences obtained herein, and those of other *Trochoidea* species segments deposited in GenBank, produced similar trees (Figure 3). T*rochoidea* samples were grouped into two main clades: one included *T. pyramidata* and *T. trochoides*, each of which was grouped in turn in two distinct subclades; the other including the subclades *T. elegans* and *T. caroni*, and the distant subclade *T. spratti*. In *T. caroni*, the samples from Palermo were external to the subclade, including samples from Trapani, Capri, and Terracina, with the last two samples showing a unique haplotype (Figure 3).

## 4. Discussion

*T. caroni* is known to occur in central-western Sicily and Capri, and because of this, Bodon et al. [5] proposed that the population on Capri was a relict population of a wider peninsular species distribution, while its presence on the island, or even in the Italian peninsula, was due to human activity [6,32].

**Figure 4:**
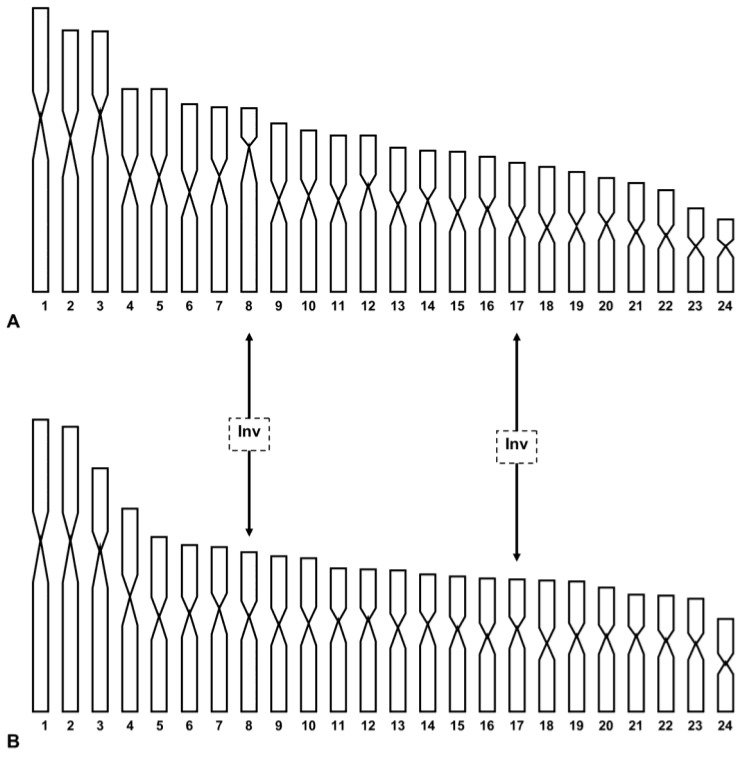
Haploid karyograms of *T. caroni* from Capri (**A**) and Palermo (**B**), showing the origin of chromosomal differences between them on the basis of the inversion hypothesis. Inv = inversion.

New discoveries of live specimens or their shells in continental Italy support a wider distribution of *T. caroni* than originally thought [6]. The results obtained in this study show that the continental and Sicilian populations are karyologically differentiated due to the different morphology of pairs 8 (subtelo- vs. metacentric) and 17 (metacentric vs. submetacentric) in the Capri and Palermo populations, respectively (see Figure 1 and Table 1). We put forward the hypothesis that the chromosomal rearrangements involved were two inversions or centromeric shifts [33] (see Figure 4). Other mechanisms, such as translocations or heterochromatin addition/deletion, could be involved. Chromosome painting could detect translocation events, while we maintain that the occurrence of heterochromatin addition/deletion is improbable, as two populations have a very scarce centromeric heterochromatin (authors’ observations; see also [11,14]).

Chromosomal polymorphism due to inversions and their role in the speciation process are matters of debate [34]. However, accumulating evidence shows that chromosomal polymorphism (such as inversions, chromosome fusions or fissions, and translocations), by reducing recombination between favourable combinations of alleles, is an important driving force in local adaptation, speciation processes, and sex chromosome evolution in both animals and plants [35,36].

Regarding the chromosomal diversification between the two populations of *T. caroni* studied, our preliminary molecular analysis shows *T. caroni* to be closely related to *T. elegans* and *T. spratti* as its sister species. Furthermore, 16S rDNA analysis shows that the populations of *T. caroni* from Capri and Terracina have a unique haplotype, separate from the Sicilian populations (p-distance 0.12), which in contrast display a discrete degree of diversification (p-distance 0.12). On the other hand, diversification among mainland Italy and Sicilian populations is well known and documented for different taxa, both vegetables (e.g., oak populations) [37]) and animals (e.g., Pulmonata gastropods of the genus *Marmorana*, anurans of the genus *Bufotes*, rabbits of the genus *Oryctolagus*, and butterflies of genera *Pieris*, *Iphiclides*, and *Anthocharis*) [38,39,40,41].

In the Italian peninsula and Sicily, a combination of geological–paleoclimatic events occurred (the salinity crisis in the Miocene; the glacial and interglacial periods in the Pliocene; and in the Pleistocene), resulting in population isolation and differentiation, dispersal/vicariance phenomena, introgression, hybridization, extinction, and other speciation processes with a high level of endemism [42,43,44,45,46]. However, further karyological and molecular studies are needed to provide insights into the degree of diversification between the continental and Sicilian populations of *T. caroni*.

It should be noted that species of the “*elegans*” group (*T. elegans*, *T. caroni*, *T. spratti*, and *T. cumiae*) to which *T. caroni* belongs have very similar internal anatomy (genitalia), and their distinction is mainly based on geographic distribution and their remarkable shell morphology [47,48]. According to Giusti et al. [7], the specific validity of “*elegans*” group members requires confirmation.

The karyotypes of *T. caroni* here described differ from those of *T. elegans* and of *T. pyramidata* and *T. trochoides*, the other species in the genus [11]. In fact, all *Trochoidea* so far studied have 2*n* = 48, but with a different chromosomal formula: *T. caroni* has 19M, 4sM, 1sT (from Capri), and 19M, 5 sM (from Palermo); *T. elegans* has 16M, 6sM, 2T; *T. pyramidata* has 20M, 4sM (from Capri); and *T. trochoides* has 16M, 7sM, 1T [11]. For the chromosome formula of *T. pyramidata*, we report the origin (Capri) and GenBank accession number (MZ504248-50) of the samples studied by [11], because the studied Capri samples were genetically different to the specimens from Tunisia (AN: KY747545); Siena, Italy (AN: AY741444); Italy (AN: KU521590); and Spain (AN KJ458565, AN: AY546377) [10,18,30,49], highlighting that *T. pyramidata* populations require taxonomic revision.

The *Trochoidea* clade appears to be karyologically uniform in terms of numbers of chromosomes (2*n* = 48). This could also apply to the NOR loci that are located on medium-sized chromosome pairs in both *T. caroni* and *T. elegans* (see Figure 2).

However, similar localisation of the NORs is displayed from the closely related species *Cernuella virgata*, of the tribe Cernuellini [11], suggesting that the NOR loci on a medium-sized chromosome pair (tentatively, the 16th pair) were inherited from the common ancestor of Trochoideini + Cernuellini.

The karyological data of the Trochoideini, i.e., of the genus *Xerocrassa*, concern only the number of chromosomes (2*n* = 52 in *X. cretica*, and 2*n* = 50 in *X. geyer*) [50,51]. Among the Cernuellini species, a chromosomal formula was described in *C. virgata*, which conserved the presumed primitive Geomitridae karyotype of 2*n* = 52 with all metacentric chromosomes [11].

On this basis, the 2*n* = 48 primitive karyotype of the common ancestor of *Trochoidea* could have originated from the translocation of two pairs of chromosomes of the primitive Geomitridae complement of 2*n* = 52 chromosomes. In Figure 4, we advance a hypothesis of the chromosome evolution that occurred during the specific diversification of *Trochoidea*. The hypothesis deserves confirmation from further chromosomal analysis performed by banding and molecular methods.

**Figure 5:**
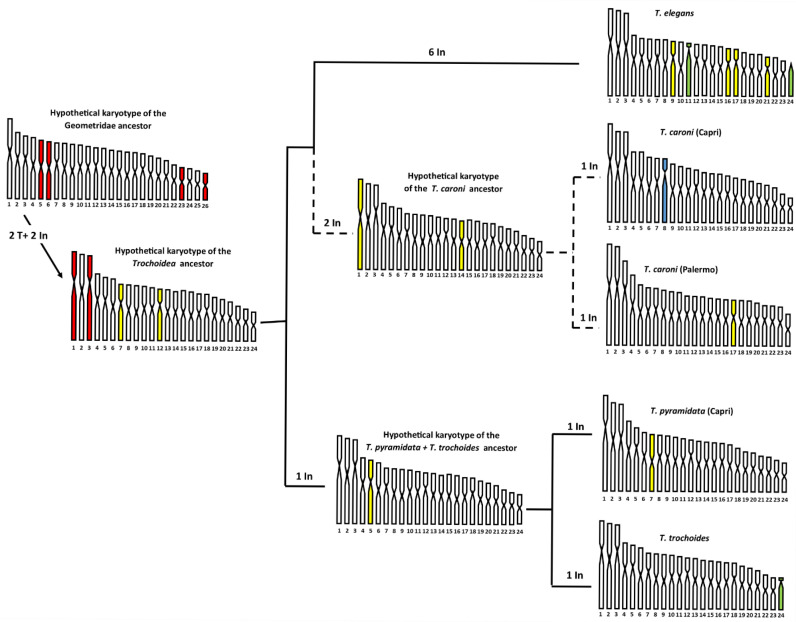
Hypothetical chromosome evolution of the *Trochoidea*. Phylogenetic relationships between *T. elegans*, *T. pyramidata*, and *T. trochoides* are represented according to Razkin et al. [10] (branch lengths do not respect genetic distance). The relationships between *T. elegans* and *T. caroni* take into consideration that the species belong to the *T. elegans* group. The chromosomes involved in the rearrangements are highlighted in various colours (see the test for further explanation). T = translocation; In = inversion.

Note that the presumed primitive karyotype of the common ancestor of *Trochoidea* was derived by considering the most common form of chromosomes between the karyotypes of *T. caroni* from Capri and Palermo, presented here, and those of *T. elegans*, *T. pyramidata*, and *T. trochoides* [11], whose relationships place *T. elegans* sister to *T. pyramidata* + *T. trochoides* clade, according to Razkin et al. [10]. It should be emphasised that in representing the presumed primitive karyotype of *Trochoidea* we have not retained the chromosome order assigned by Petraccioli et al. [11] in the karyotype of *T. elegans*, *T. pyramidata*, and *T. trochoides*. In fact, to respect the chromosome shape, in some cases we shifted the original chromosome order to a nearby position, but always considering the standard deviation values. This was done to minimise rearrangements, then using parsimony. In our hypothetical scenario (Figure 5), we assume that the primitive karyotype of *Trochoidea* is derived from the translocation of two pairs of chromosomes, tentatively, pairs 23 and 26 on pairs 5 and 6 of the primitive karyotype of Geomitridae of 2*n* = 52 (filled in brown), giving rise to the submetacentric pairs 1 and 3 of 2*n* = 48 of the karyotypes of the common *Trochoidea*. As a result of translocations, the formation of neo-centromeres also occurred [52,53], as well as two inversions, which involved the original metacentric pairs 7 and 12 of the primitive Geomitridae karyotype, and transformed them into submetacentric pairs in the common karyotype of *Trochoidea* (filled in yellow). Several chromosome inversions occurred in this primitive karyotype during the specific diversification of the genus. In particular, in the *T. pyramidata* + *T. trochoides* clade there was first an inversion that led to the submetacentric shape of the original chromosome 5 of their common ancestor (filled in yellow in Figure 5), then a further inversion occurred in both the karyotype of *T. pyramidata* and *T. trochoides*, involving, respectively, pair 7 and the last pair, making them metacentric in *T. pyramidata* and telocentric in *T. trochoides*, highlighted in yellow and green in the relative karyotypes in Figure 5.

In the *T. elegans* + *T. caroni* clade, the first two inversions involved pairs 1 (submetacentric) and 14 (metacentric) of the common progenitor of *Trochoidea*, and these pairs were, respectively, metacentric and submetacentric in the common progenitor karyotype of the Capri and Palermo populations of *T. caroni*. In the last two populations, a further inversion occurred, with the chromosomes of pair 8 of the Capri shaped as subtelocentric and the chromosomes of pair 17 of the Palermo shaped as submetacentric (highlighted, respectively, in light blue and yellow in Figure 5).

Finally, the karyotype of *T. elegans* is derived from that of the common ancestor of *T. trochoides* by six inversions. Four of them formed as submetracentric chromosomes, pairs 9, 16, 17, and 21 (highlighted in yellow in the relative karyotype of Figure 5). The other two inversions formed as telocentric, pairs 11 and 24 (highlighted in green in the relative karyotype of Figure 5).

## 5. Conclusions

In conclusion, we first described the karyotype of the land snail *T. caroni*. It has 2*n* = 48 mostly bi-armed chromosomes, but Sicilian populations karyologically differ from the Capri and Terracina populations in the morphology of two pairs of chromosomes, probably originating from inversions. Preliminary molecular analysis also evidences a discrete diversification between the Sicilian population and those outside of Sicily. Comparison with data available in the literature on *T. caroni* relatives (*T. elegans*, *T pyramidata* and *T. trochoides*) allowed us to define the probable karyotype of the common ancestor of the *Trochoidea* (2*n* = 48, all biarmed chromosomes), which in turn was derived from the 2*n* = 52 all-metacentric primitive karyotype of Geomitridae by means of two translocations and two inversions. In addition, a hypothesis has also been put forward suggesting that during the specific diversification of *Trochoidea*, chromosomal evolution occurred through intrachromosomal rearrangements, and that NOR loci were conserved on chromosomes of a medium-sized pair in both Trochoideini and Cernuellini snails.

## Figures and Tables

**Table 1:** Morphometric parameters of the chromosomes of *T. caroni* samples from Capri and Palermo. R.L. = relative length (chromosome length/total chromosome length × 100); C.I. = centromeric index (short arm/chromosome length × 100); Sh (chromosome shape; M = metacentric, sM = submetacentric, sT = subtelocentric).

	*T. caroni* (Capri/Terracina)			*T. caroni* (Palermo)		
Chromosome	R.L.	C.I.	Sh	R.L.	C.I.	Sh
1	7.5 ± 0.8	39.0 ± 2.4	(M)	7.7 ± 0.7	39.8 ± 2.9	(M)
2	6.8 ± 0.4	40.9 ± 1.7	(M)	7.5 ± 0.6	41.9 ± 2.6	(M)
3	6.8 ± 0.6	32.3 ± 1.2	(sM)	6.3 ± 0.7	35.1 ± 2.0	(sM)
4	5.3 ± 0.3	43.3 ± 1.5	(M)	5.3 ± 0.5	42.5 ± 2.7	(M)
5	5.3 ± 0.7	42.8 ± 1.0	(M)	4.6 ± 0.4	40.8 ± 2.3	(M)
6	4.9 ± 0.5	46.9 ± 2.9	(M)	4.4 ± 0.6	46.3 ± 2.5	(M)
7	4.8 ± 0.3	36.2 ± 2.0	(sM)	4.3 ± 0.8	36.2 ± 2.6	(sM)
8	4.8 ± 0.8	22.4 ± 2.5	(sT)	4.2 ± 0.5	40.2 ± 2.0	(M)
9	4.4 ± 0.2	44.9 ± 2.7	(M)	4.1 ± 0.6	43.8 ± 2.4	(M)
10	4.2 ± 0.7	41.2 ± 2.2	(M)	4.0 ± 0.8	41.9 ± 2.2	(M)
11	4.1 ± 0.8	40.3 ± 1.8	(M)	3.8 ± 0.4	41.8 ± 2.9	(M)
12	4.0 ± 0.6	34.3 ± 2.1	(sM)	3.8 ± 0.4	35.4 ± 1.7	(sM)
13	3.8 ± 0.6	39.5 ± 1.7	(M)	3.7 ± 0.5	39.5 ± 2.0	(M)
14	3.8 ± 0.4	33.6 ± 1.6	(sM)	3.6 ± 0.7	33.9 ± 1.6	(sM)
15	3.7 ± 0.5	43.6 ± 2.3	(M)	3.6 ± 0.6	45.4 ± 1.9	(M)
16	3.5 ± 0.8	38.9 ± 2.1	(M)	3.6 ± 0.5	39.4 ± 2.4	(M)
17	3.4 ± 0.4	44.1 ± 2.0	(M)	3.5 ± 0.6	35.1 ± 2.2	(sM)
18	3.3 ± 0.5	48.3 ± 1.5	(M)	3.5 ± 0.9	49.1 ± 2.1	(M)
19	3.1 ± 0.6	45.4 ± 2.6	(M)	3.5 ± 0.7	43.0 ± 2.7	(M)
20	2.9 ± 0.5	40.2 ± 3.1	(M)	3.3 ± 0.3	39.7 ± 2.0	(M)
21	2.8 ± 0.5	44.9 ± 2.9	(M)	3.1 ± 2.2	35.8 ± 2.3	(M)
22	2.7 ± 0.4	43.5 ± 2.5	(M)	3.1 ± 2.0	40.3 ± 2.7	(M)
23	2.3 ± 0.3	43.5 ± 2.1	(M)	3.0 ± 2.4	46.6 ± 1.9	(M)
24	1.9 ± 0.2	39.3 ± 1.9	(M)	2.5 ± 2.7	39.4 ± 2.0	(M)

## Data Availability

Newly generated cytogenetic data are available within this manuscript.
